# Community Size Effects on Epidemic Spreading in Multiplex Social Networks

**DOI:** 10.1371/journal.pone.0152021

**Published:** 2016-03-23

**Authors:** Ting Liu, Ping Li, Yan Chen, Jie Zhang

**Affiliations:** 1 Center for Intelligent and Networked Systems, School of Computer Science, Southwest Petroleum University, Chengdu 610500, China; 2 Center for Computational Systems Biology, Fudan University, Shanghai 200433, China; University of Waterloo, CANADA

## Abstract

The dynamical process of epidemic spreading has drawn much attention of the complex network community. In the network paradigm, diseases spread from one person to another through the social ties amongst the population. There are a variety of factors that govern the processes of disease spreading on the networks. A common but not negligible factor is people’s reaction to the outbreak of epidemics. Such reaction can be related information dissemination or self-protection. In this work, we explore the interactions between disease spreading and population response in terms of information diffusion and individuals’ alertness. We model the system by mapping multiplex networks into two-layer networks and incorporating individuals’ risk awareness, on the assumption that their response to the disease spreading depends on the size of the community they belong to. By comparing the final incidence of diseases in multiplex networks, we find that there is considerable mitigation of diseases spreading for full phase of spreading speed when individuals’ protection responses are introduced. Interestingly, the degree of community overlap between the two layers is found to be critical factor that affects the final incidence. We also analyze the consequences of the epidemic incidence in communities with different sizes and the impacts of community overlap between two layers. Specifically, as the diseases information makes individuals alert and take measures to prevent the diseases, the effective protection is more striking in small community. These phenomena can be explained by the multiplexity of the networked system and the competition between two spreading processes.

## Introduction

Diseases spreading in a population takes place via the interactions of infected individuals with others. Despite the complexity of contact pattern and individuals’ behavior, much efforts have been devoted to modeling the diffusion of pathogens in network science as mentioned in [1]. In recent years, the epidemic spreading processes on single contact networks have been studied [2–5]. However, with rapid development of information technology, many hi-tech products come into our daily life, which makes people contact with others not solely in real-life but also in online social networks, leading to extensive information exchange across geographic boundaries. Accordingly, not only diseases themselves but the information about diseases propagates via off-line or online social networks. Although it seems that the dynamic process of epidemic spreading separates from the information spreading, but when the entities in these two processes are the same, disease spread might be affected by information diffusion. For example, during the flu season, a person got influenza he put a message on Facebook or Twitter about his flu. His friends would reduce their risk to be infected by the man by taking a flu shot or avoiding contacting the man if they got this information. With regard to many realistic scenarios like the above situation in real-world networks, if many susceptible individuals know these information, their protection awareness may increase, which makes people alert and even change behaviors. Thus the outbreak of infectious diseases would be controlled or even vanished.

Considering the effects of information spreading process, we simulate the dynamic spreading processes of epidemic and information on multiplex networks. In fact, the issue of spreading dynamics on multiplex networks has recently attracted considerable attentions [6–9]. For instance, Granell et al. [6] discovered the emergency of a metacritical point where the diffusion of awareness is able to control the onset of epidemics by using the multiplex network model (SIS and UAU), in their work the dynamic processes of the spreading of awareness and epidemics are the same. Guo et al. [7] used the same model as Ref. [6] but different awareness spreading process: the unaware individuals become aware depending on the local awareness ratio (the ratio between their aware neighbors and their degrees). As a survey, authors in [1] discussed various research works which have modeled spreading processes in multilayer networks and categorized existing model into two groups: epidemic-like [9, 10] and decision-based. Different from the previous works on modeling epidemic-like spreading processes with multiplex networks, here we focus on the roles played by community structure in affecting disease spreading, especially the effect of community size. To this end, we formulate the spreading processes under the framework of multiplex networks which consists of two networks with community structure: dynamical process of information spreading can be effectively considered as an online communication network; the real-world epidemic system can be considered as a physical contact network. All nodes represent the same entities in both networks, however these nodes may not have the same neighbors, i.e. the interconnection may be very different in these two networks. For example, a man who follows you in twitter can make continuous interactions with you through the social network, even though in the real-world he is far away from you so that he does not have any physical contact with you.

Some previous works on epidemic spreading [3–5, 11] have also taken the community structure of contact network into consideration. Ref. [11] implemented various local strategies which most of them are based on community structure and those strategies insist that an individual should try to avoid contact with those in different communities. Different from the existing control strategies that implement edges removal when individuals become infected and develop symptoms, we simulate the dynamic processes on multiplex networks and modify the epidemic transmission rate when the susceptible individuals got the disease information from the online social network. Once an epidemic emerges, the aware individuals tend to take some measures to avoid infection, at the same time, spread the disease information on their social network. Certainly, whether to take protecting measures or not depends on people’s disease awareness, which is determined by varied factors, such as prevalence of the disease and the heterogeneous population. In the literature of sociology, some studies [12–14] have used social network theory and metrics to examine how social structures influence social behaviors. Particularly, it is manifested that population size has an important impacts on the diseases awareness of individuals in that population [2, 15, 16]. For instance, if your schoolmates got pinkeye and there are thousands of students in your school, you may not take great care. But if one of your roommates got this disease, you must pay much attention and take actions to prevent yourself from being infected.

**Fig 1:**
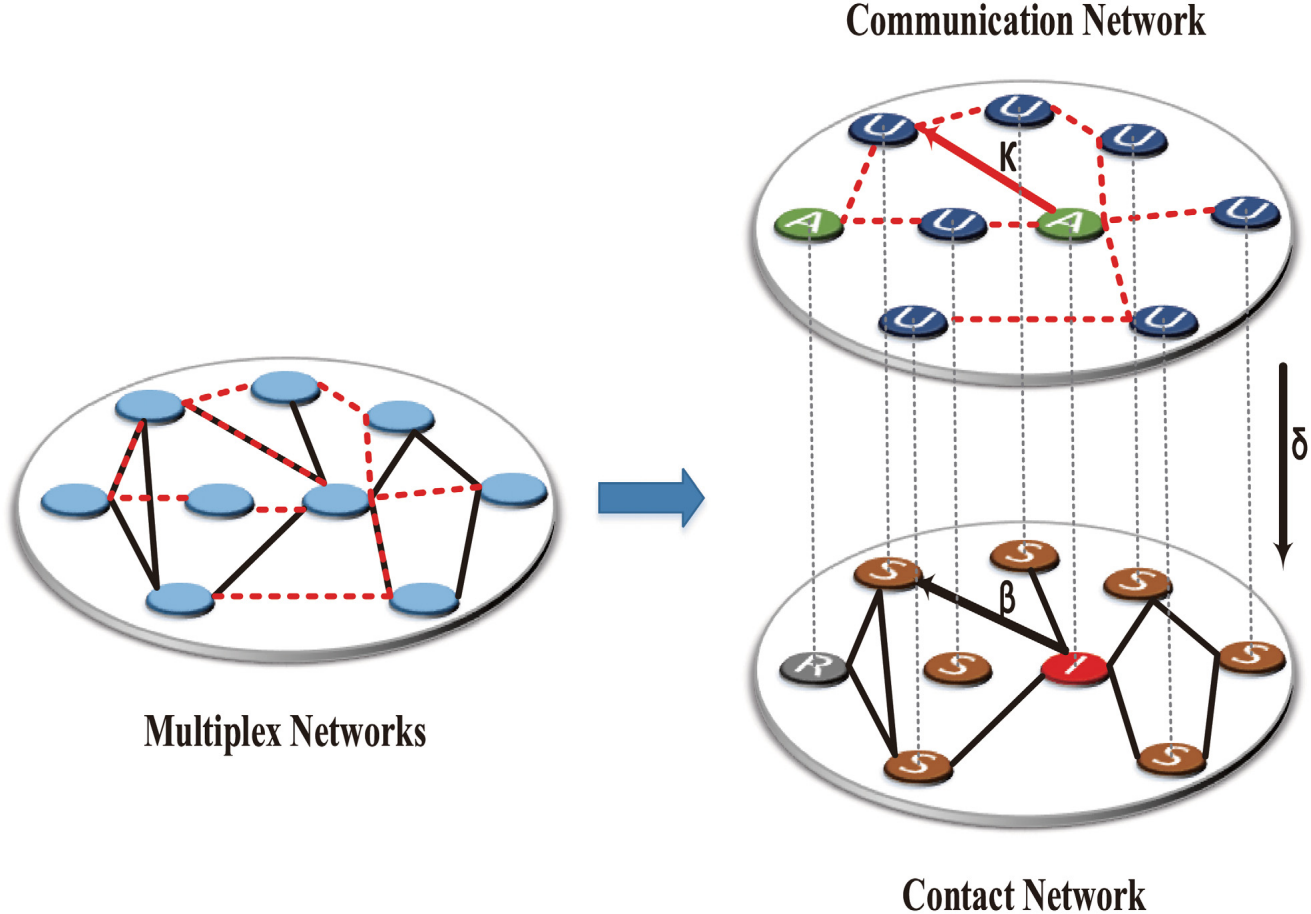
The sketch of a multiplex network used in our model. The top-right layer is supporting the spread of information in the online social network, in which nodes have two possible states: active (A) or inactive (U), where the parameter *κ* represents the activation rate. The bottom-right layer is supporting the spread of epidemic in the real-world, in which nodes have three possible states: susceptible (S) or infected (I) or recovered (R). Moreover, these nodes one-to-one map with the top layer and the parameter *β* is the infectivity rate.

Moreover, to explore how individuals’ awareness is determined by the community feature, which in turn affects the propagation of diseases, we assume that the contact network on one layer of multiplex network model is modular. That is, the contact network divides naturally into groups of nodes with dense connections internally and sparse connections between groups, which is very common in social networks [16]. We numerically study the effect of community size on individuals’ self-protection and the resulting disease incidence. We find that the epidemic spreading have an obvious change when the immunization strategy takes effect. More specifically, the final incidence of diseases is high in large subpopulations while the epidemics vanish in small subpopulations, showing the community size is influencing individuals’ immunization behavior. Furthermore, we study other aspects of community feature, i.e., the number of communities and the overlap degree of the communities between the two layers in impacting diseases’ spreading. The results show that the more communities a network has, the less the network is infected. On the other hand, the impacts of community overlap of two layers on final incidence of diseases depend on information dissemination rate and infectivity rate as well. Fig 1 shows the sketch of these two networks, the probability of the nodes activated by the disease information is represented by the parameter *κ*, and the parameter *β* regulates the probability that susceptible individuals are infected by infectious diseases. Meanwhile, the parameter *δ* depends on the population size of contact network and restrains the infectivity rate *β* only for the case in which nodes got the disease information through the communication network.

In subsequent sections, we firstly introduce the model employed and relevant spreading processes, analyze our immunization strategy. Secondly we briefly describe the network types used in our experiments. Then we detail our experiments and present an analysis of these results. Finally, we make our concluding remarks. In the latter part, we detail our model and immunization strategy utilized in this paper.

## Analysis

Existing models of epidemic dynamics allow us to investigate many realistic scenarios such as population heterogeneity, social structures and mobility processes down to the individual level [17]. Much of the research on modeling the dynamics of spreading over multiplex networks has used epidemic model like Susceptible-Infected-Recovered (SIR) [18–23] and Susceptible-Infected-Susceptible (SIS) [24–26]. Here, we adopt the Susceptible-Infected-Recovered (SIR) model to depict the epidemic spreading process on the layer of contact network. On the other hand, the information about epidemic can simultaneously propagate via the other layer of the multiplex network, i.e., the communication network [27]. There are two widely accepted models that can be employed in the work, that is, threshold model of diffusion [28] and Independent Cascade (IC) model of diffusion [29, 30]. In this work, we use IC model to characterize the disease information propagation. For the sake of simplicity, we assume that the infected individuals must be aware of the disease and try to transfer the information to their neighbors in the social network. As a result, by integrating the SIR model on one layer and the IC model on the other layer of the multiplex network, a dual spreading process can readily be modeled. In the SIR-IC model, an individual can be in four states as the following: active and susceptible (AS), active and infected (AI), active and recovered (AR) or inactive and susceptible (US).

**Fig 2:**
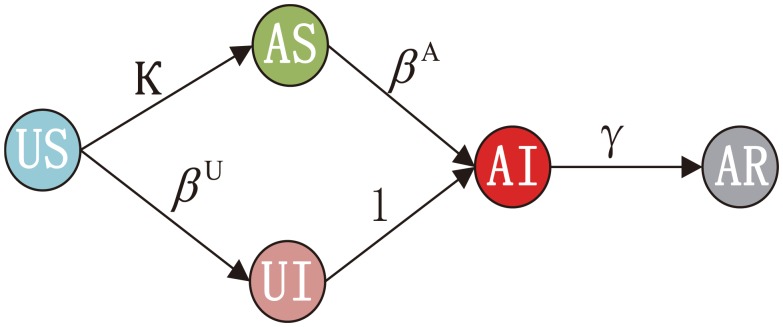
All possible states of the individuals in the multiplex network. AS represents active and susceptible individuals. Likewise, AI represents active and infected individuals, AR denotes active and recovered individuals, US indicates inactive and susceptible individuals, other states: inactive and infected (UI) transition to active and infected (AI) automatically. The transition probabilities are *κ*, *β*^*A*^, *β*^*U*^ and *γ*, respectively.

Initially, all individuals are susceptible and inactive (US) and a few individuals randomly chosen nodes from the multiplex network to be infected (AI), which means these nodes are infected on the contact network layer and are in active state on the communication network layer. Then epidemics and information spread on different layers of the multiplex network with evolution rules given by SIR and IC models, respectively. We note that the infected will immediately become active, implying that the infected individuals are automatically aware of the risk of disease and will help with dissemination of the disease information. Consequently, the information diffusion process is influenced by epidemic spreading process. At each time step, individuals in susceptible state will be infected by their infected neighbors with probability *β*. However, the information diffusion makes a feedback on epidemic spreading when the activated individuals (informed and convinced the information) take protective measures to reduce the probability of being infected. It should also be noted that at each time step in information diffusion process described by IC model, active individuals have a single chance to transmit the disease information to each of their inactive neighbors with probability *κ* as shown in Fig 2. Whether or not the transmission succeeds, these active individuals cannot make any further attempts to influence the same neighbors. If the transmission succeeds, their neighbors will become active and never change to inactive [31]. With respect to epidemic spreading process, there are certain numbers of nodes in three states at time t, denoted by *S*(*t*), *I*(*t*), *R*(*t*), respectively. Additionally, *s*(*t*), *i*(*t*), *r*(*t*) represent the fractions of each state, respectively. At each time step, these numbers must satisfy the fixed equation: *N* = *S*(*t*) + *I*(*t*) + *R*(*t*), where *N* is the total number of the individuals of the network. Meanwhile, to measure the incidence of diseases, the order parameter *ρ*^*I*^ is given by the following equation:

$$\begin{array}{c} \rho_{t}^{I}=\frac{\left(I(t)+R(t)\right)}{N} \end{array}$$ (1)

Once infected, each individual in infected state (AI or UI) can be removed from the disease to become recovered (AR) with a probability *γ*. Over time the epidemic and information spread through the multiplex network, the diffusion process terminates and the multiplex system goes into a steady state where the individuals are either susceptible or recovered. Finally, we assume that the rate of infection and recovery is much faster than the time scale of births and deaths and therefore, these factors are ignored in this model.

In our model, the individuals do not know the whole network structure and the disease states of their neighbors. Instead, we assume that each individual knows the size of community he (she) belongs to. Once the ignorant individuals are aware of disease spreading (corresponding to the active state), the crisis awareness often makes them take preventions which will reduce the possibility to be infected. It seems practical because the susceptible individuals who are aware of epidemics often make some risk-averting behaviors to avoid contacting with others and take some effective measure, e.g. take a vaccination. In general, how strong the sense of crisis an individual has is intimately relevant to the size of the community he belongs to, as aforementioned. We believe this is realistic that people who locate a large group has lower crisis awareness than whose in a small group because of the fluke mind. To incorporate the negative effect of community size on people’s vigilance to disease spreading into the system, we let the parameter *δ* denote the degree of one’s neglect about the epidemic, which depends on the community size and can be formulated as follows:

$$\begin{array}{c} \delta_{j}={\left(C_{j}\right)}^{w}. \end{array}$$ (2)

Where *C*_*j*_ is a normalization of community size *c*_*j*_ of an individual *j*, *w* is used to tunes the intensity of community size effect. Here we take *w* = 2. We also note that any other monotonic functions can also be the choice. Then (1−*δ*) represents the probability of taking immunization strategies. Accordingly, the infectivity rate is the combination of the natural infectivity of a disease and the probability of one’s neglect. Here we use two parameters to distinguish between the original unaware infectivity *β*^*U*^ = *β* and the subsequent infectivity after being aware of the epidemics *β*^*A*^ = *δβ*. From Eq (2), we can see if the active and susceptible (AS) node *j* in a very small community, the probability *δ*_*j*_ < <1, then *β*^*A*^ approximates to 0, which means the complete immunization. Note that, when *δ* = 1 and *κ* = 0, the effect of awareness is disabled and the two spreading processes evolve independently. Then two types of susceptible nodes (US and AS nodes) will be infected with probability *β*^*U*^ and *β*^*A*^, respectively, as shown in Fig 2. Moreover, the infected (AI and UI nodes) can be removed from the disease to became recovered (R) with the probability *γ*. Therefore, the effective infection rate λ is represented by $\lambda =\frac{\beta }{\gamma }$. In our experiments, we fix *γ* = 0.2 as in [11].

Next, we explore the interplay between disease information diffusion and epidemic spreading, and we specifically focus on the role of immunization awareness of individuals in the communities with various sizes. To do this, we simulate the spreads of epidemics on the SIR-IC model and compare its results with the model SIR. Then we address the comparison between a setup with community structure vs. without communities. Moreover, we explore what the roles the community size played in the spreading of diseases and how the number of communities in which the network is divided affects the stationary fraction of diseased nodes. Finally we show that the results depend on how the communities between the two layers overlap with each other in our model.

## Experimental Results

In this section, we perform numerical experiments and provide an in-depth analysis of community structure and its consequence on disease spreading.

### Network Data

To better understand the effects of information spreading and how the incidence of the epidemics is affected by the community structure of two layers, we investigate the effects of several key factors of the model: infection rate, activation rate, the degree of neglect about the epidemic and other community features like community number and the overlap of communities of two layers. To this end, we create multiple networks for different experiments where individuals are represented by the vertices and their contacts are represented by edges. The networks used in the experiments are given as following:

Both epidemic spreading layer and communication layer of multiplex networks are constructed by the “benchmark” algorithm proposed by Lancichinetti, Fortunato, and Radicchi (LFR) [32], with which the networks with community structures and power-law distribution of community size can be generated. In our experiments, the exponents of community size distribution are set to be 2 for epidemic spreading layer and 2.5 for information diffusion layer, respectively. Network size *N* ranges from 1000 to 20000 and the average degree is identical for the same layers of all multiplex networks throughout this paper. In addition, null model is used to generate networks without community structure for comparison. This can be realized by randomly rewiring the networks generated from LFR algorithm while keeping the degree sequences [33].

To explore the effect of community number a network has on compound spreading dynamics, we change the community number of contact networks using the algorithm presented in [34] while preserve the network properties unchanged. Another factor related to community structure is the overlap of community members between two layers. In fact, it is possible for two layers that some of their communities have members in common. To get insight into the role of overlap degree of two layers’ communities, we generate the communication network by permuting the community labels of the nodes in contact network with probability *π* (*π* varies between 0 and 1). This way, one can adjust the community overlap degree between two layers without changing any network properties. In order to distinguish with other networks, we use LFR-V to symbol this type of artificial networks.

### Interplay between epidemic spreading and information diffusion

**Fig 3:**
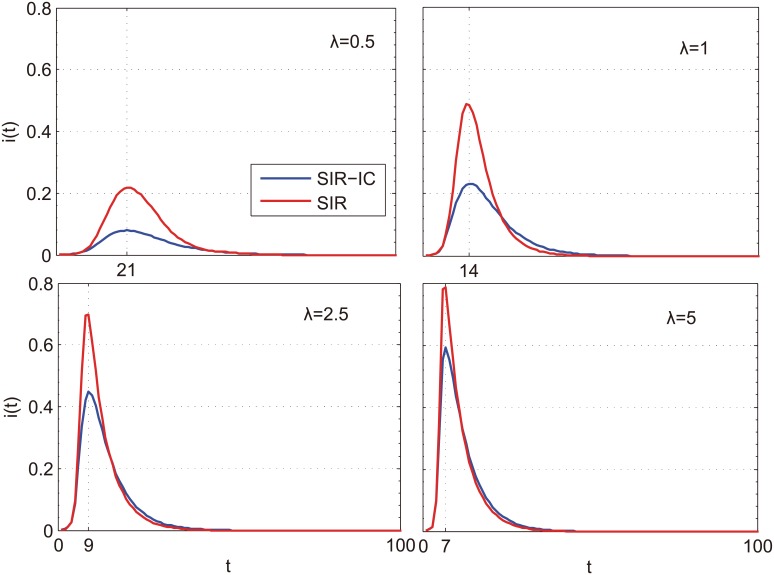
Comparison of the infected individuals fraction i(t) using single SIR model (red line) simulation and add the IC model (blue line) simulation as a function of the time step *t* for different values of parameter λ. The structures of the networks in this experiment are the same and the rest of the values of parameters are: *κ* = 0.1, *γ* = 0.2, *N* = 1000, <*k* > = 14 for epidemic spreading layer and <*k* > = 16 for information diffusion layer. The community size of epidemic spreading layer ranges from 10 to 96.

For a multiplex networked system, it is of particular interest to understand how different dynamical processes in different layers interact with each other. To this end, we implement both SIR-IC and SIR models for different values of parameters *β* and *κ* on LFR networks, and then compare incidence in the two models. The infected ratio *i*(*t*) of SIR and SIR-IC models over time is illustrated in Fig 3. By comparing the four panels, it is clear that there is a significant change on the peak of *i*(*t*) and the infected speed with λ. As shown in all subplots of Fig 3, the peak of the curves goes up more and more quickly with the increase of the effective infection rate λ. By contrast, the peak of *i*(*t*) in each panel is always much higher in SIR model than in SIR-IC model (corresponding to the blue line). It is manifested from the results that the effects of awareness are considerable even the information spread slowly (*κ* = 0.1) in the communication network.

**Fig 4:**
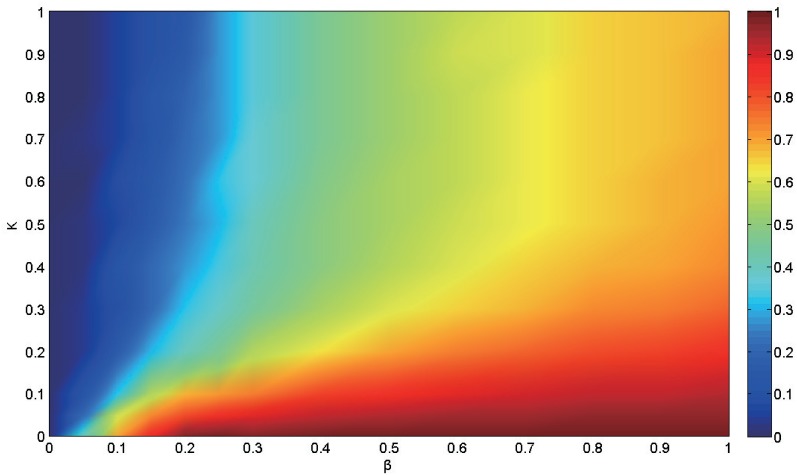
The full phase diagram of the fraction of diseased individuals *ρ*^*I*^ in the stationary state(color represent the fraction value). Here, *γ* = 0.2, *N* = 1000.

To further explore the effects of the information spreading, we explore the full phase of the SIR-IC model. Fig 4 shows the infection incidence in the stationary states under different conditions. It is revealed from Fig 4 that for very small activation probability *κ* the incidence of the epidemic is relatively large, in regardless of the disease infection rate, which corresponds to the red area close to the horizontal axis. The reason is straightforward: when the information diffuses slowly in the communication network, only a few nodes are aware of the epidemic and then can take preventive strategies, which exerts very limited impact on epidemic spreading. Thus, the resulting infection incidence is less relevant to the activation rate and it is easier for outbreak of epidemics. However, in the case of lower infection rate the epidemic spread can be suppressed by the information diffusion process, as shown in the area close to the vertical axis in Fig 4. The competition between the two processes is more prominent for parameters within the central area of the parameter space. Furthermore, as we can see from Fig 4, the blue area (corresponding to the phase that the epidemic does not propagate) expands with the increase of activation rate when the infection rate has low values. That is, increasing the information activation rate enhances the epidemic threshold. This phenomenon can be explained as follows: the large value of activation rate causes most individuals to aware the disease information and take protection measures, which therefore decreases the infection rate and increases the threshold.

**Fig 5:**
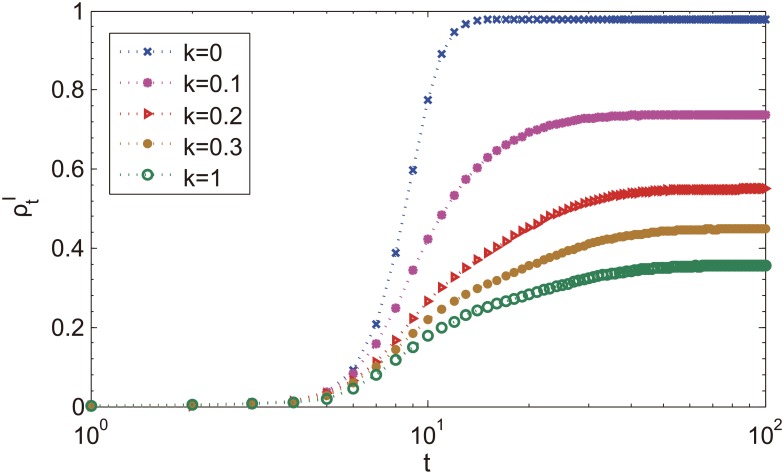
The fraction of diseased individuals $\rho_{t}^{I}$ in SIR-IC system as a function of the time *t* for different values of activation rate *κ*. *κ* = 0: diseases information are not taken into account in this case. We fix other parameters as follow: *β* = 0.3, *γ* = 0.2 (λ = 1.5), *N* = 1000.

**Fig 6:**
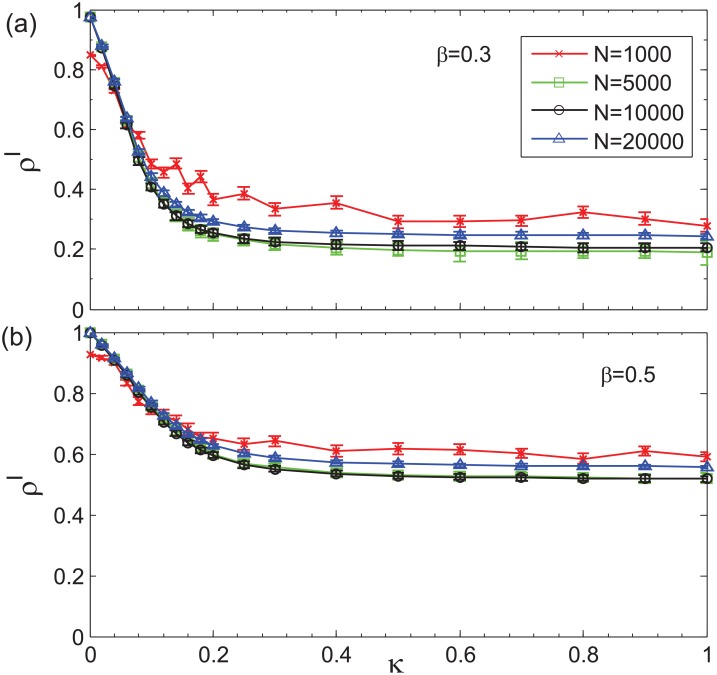
The comparison of final incidence *ρ*^*I*^ using different multiplex networks as a function of activation rate *κ*. The two layers of multiplex networks both are LFR networks and we simulate our model at least 150 times on those networks with different sizes *N*. We fix other values of parameters are: *γ* = 0.2, <*k* > = 14 for epidemic spreading layer and <*k* > = 16 for information diffusion layer. (a) Infection rate *β* = 0.3. (b) *β* = 0.5.

Now we detail the process of epidemic spreading: the enhancement of the epidemic size over time is obvious in Fig 5. At the first few steps, $\rho_{t}^{I}$ for different *κ* evolve in a similar way, while in the stationary time steps, gaps emerge among the curves, showing the decrease in the number of infected individuals for larger parameter *κ* > 0. Thus it is concluded from Fig 5 that the information spreading of disease has a significant impact on the dynamic of epidemic spreading. The reason for the reduction in diseased individuals roots in the generation of immunization awareness and effective behavioral changes, which leads to a smaller exposure of susceptible individuals to the infected population. As to the final incidence, the fraction of diseased individuals is much lower than the case without information spreading (*κ* = 0). In Fig 6, we report the fraction of diseased individuals at the end of epidemic spreading as a function of different *κ* for different network size *N* with error bars. It is shown that the final incidences are restrained with increasing *κ* at low values of *κ*, but independent of *κ* at high values of *κ*. This can be explained as following: when the activation rate *κ* is enough high, most of the individuals will be active at the first few steps and the number of active individuals varies slightly even increase the activation rate. As a result, the final incidences keep constant.

### The Effects of Community Structure

The experimental results have shown the interdependence of the epidemic spreading and the information diffusion in the multiplex networks, which is attributed to the individuals’ response to the epidemic spreading. In this part, we further study the role of community structure in spreading processes. We compare the final incidences of two types of multiplex networks. In the first type of multiplex networks, the contact network layer has the property of community structures while in the second type of multiplex networks the contact network layer is randomly connected. To test this, we separately run the spreading processes on top of multiplex networks whose contact network is generated by *LFR* or null model.

Note that the community structure leads to individuals’ neglect about the epidemic (as shown in Eq (2)), the actual infectivity rate for informed nodes is *β*^*A*^. However, for the contact networks that are completely random, each node will not belong to any groups or communities, which in fact is an ideal model. Therefore, Eq 2 cannot be used to calculate the infectivity rate in this case. In view of this, we regard each node as a community and suppose that the degree of one’s neglect about the epidemic is identical for all nodes and smaller than those of modular contact networks. Because usually one will raise his vigilance when he (she) is alone. Here we let the parameter *δ* to be 0.01.

**Fig 7:**
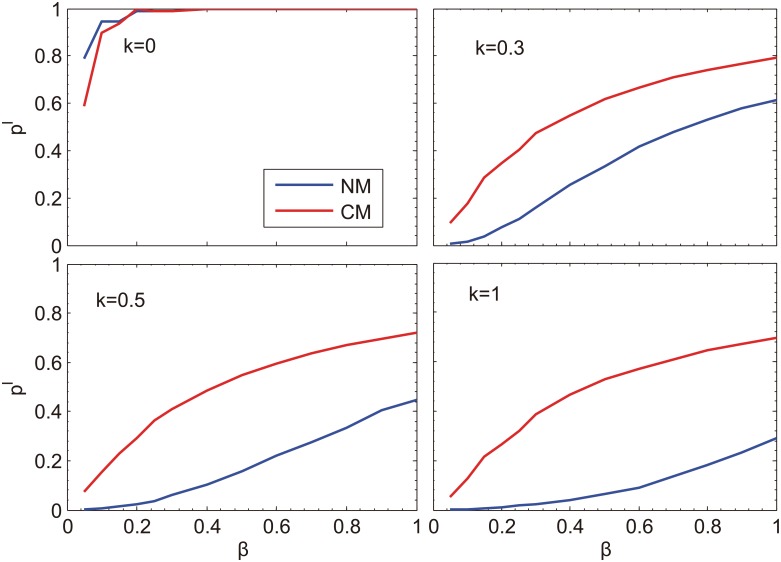
Comparison of the final incidence *ρ*^*I*^ of diseases for two types of multiplex networks. NM corresponds to the case that the contact network is randomly connected in SIR-IC, while CM corresponds to contact networks with community structure. The results for different activation rate *κ* are shown in four subplots, respectively. For all cases, *γ* = 0.2, *N* = 1000.

These experiments are implemented on many realizations of the network models for several activation rates. The results are shown in Fig 7. It can be seen that although the incidence is higher in the multiplex networks with random contact layer than in the multiplex networks with community contact layer when there is no information being diffused (corresponding to *κ* = 0), random contacts are manifested to be more effective than community organizations in restraining epidemic spreading when the disease information is available.

### The Effect of Community Size

**Fig 8:**
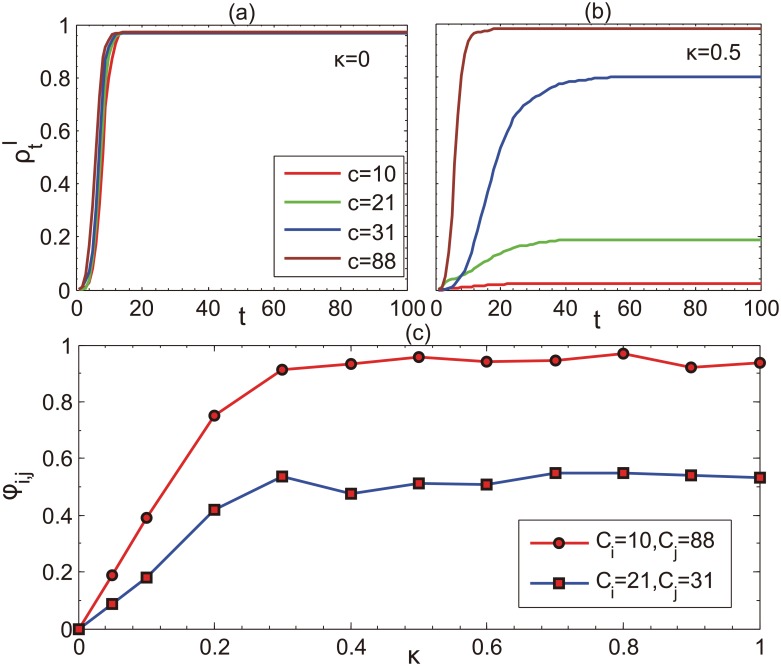
Comparison of the fraction of diseased individuals $\rho_{t}^{I}$ when *κ* = 0 and *κ* = 0.5 ((a),(b)). Different color lines stand for different community size c in the network. Furthermore, we also illustrate the incidence difference *ϕ*_*i*,*j*_ between community *C*_*i*_ and community *C*_*j*_ for different parameter *κ* in panel (c). The network size of two layers both are 1000. We fix the other two parameters as: *β* = 0.3, *γ* = 0.2.

According to Eq (2), the community size determines the degree of one’s vigilance and therefore the infectivity rate. Here we look into the communities to inspect the infection incidence of each community with respect to their community size. The incidence for one community is defined as the ratio of being infected and recovered in that community. Fig 8 shows the evolution of the incidence in different communities with different sizes. Comparing with the case without information diffusion, the incidences for small communities are remarkably smaller than those for larger communities, due to the negative effect of the community size on immunization. It is noteworthy that for different information diffusion rates, the incidence difference between community *i* and community *j* denoted by *ϕ*_*i*,*j*_ increases with the increase of diffusion rate at the small values, while for relatively large diffusion rates *ϕ*_*i*,*j*_ remains unchanged. This can be explained by considering the interplay between epidemic spreading process and information diffusion occurring on the two layers of the multiplex network. For small diffusion rates, epidemic spreading will infect the nodes before the nodes get information and take measures, this process is faster than information diffusion. Consequently, the community incidence rates are similar to each community. On the contrary, information diffusion overwhelms epidemic spreading with large diffusion rates. When the diffusion rate is greater than some specific value, the message will be by rapidly informed to all the nodes in the network and therefore the community size plays a role for all the communities. In this case, the resultant community incidences and their differences will keep constant.

**Fig 9:**
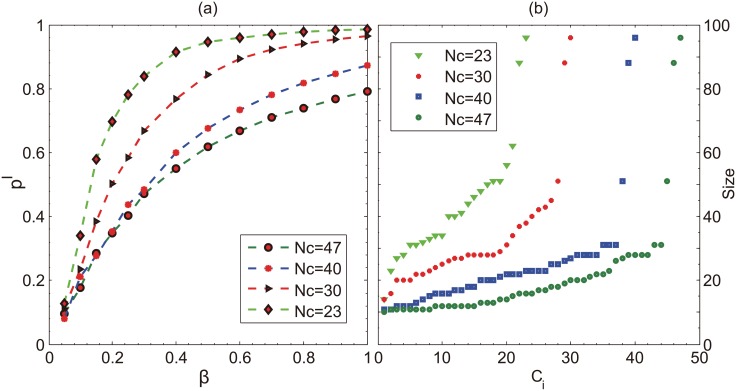
(*a*) The final incidence of disease *ρ*^*I*^ as a function of *β* for different number of communities (Nc). Other parameters are *N* = 1000, *κ* = 0.3 and *γ* = 0.2, respectively; (*b*) The size distribution of each community in the contact network with different number of communities.

Another factor related to the community effect is the number of communities a contact network has. Therefore, we implement the spreading processes on LFR networks and try different number of communities of contact network. Then we look at the final incidence for different infectivity rate. Fig 9(a) shows the positive correlation between the infection incidence and the number of communities. The reason lies in the distribution of community size (shown in Fig 9(b)). Specifically, more communities result in the decrease of the average community size, which further leads to the increase of infectivity. It should also be noted that for very small infectivity rate, the community size effect is not significant (as shown in Fig 9(a)), accordingly the incidence rates are very similar for the cases with different number of communities.

### The Effect of Communities’ Overlap between Two Layers

Since most real-world networks including the contact networks and online social networks (communication network) have been found to have community structure, it is interesting to ask how the overlap of the communities belonging to different layers affects spreading processes. To this end, we first introduce the measure to quantify the degree of overlap between the communities respectively belonging to two layers. Denote by $C^{D}\left\{C_{1}^{D},C_{2}^{D},\ldots ,C_{n}^{D}\right\}$ and $C^{I}\left\{C_{1}^{I},C_{2}^{I},\ldots ,C_{n}^{I}\right\}$ the community sets that separately belong to the contact network and the communication network. The communities’ overlap between two layers can then be formulated as follows:

$$\begin{array}{c} Q=\frac{1}{n}\sum_{i=1}^{n}max_{C_{j}^{I}\in C^{I}}\left(\frac{C_{i}^{D}\cap C_{j}^{I}}{C_{i}^{D}\cup C_{j}^{I}}\right). \end{array}$$ (3)

Where the symbol *n* is the number of communities a contact network has.

**Fig 10:**
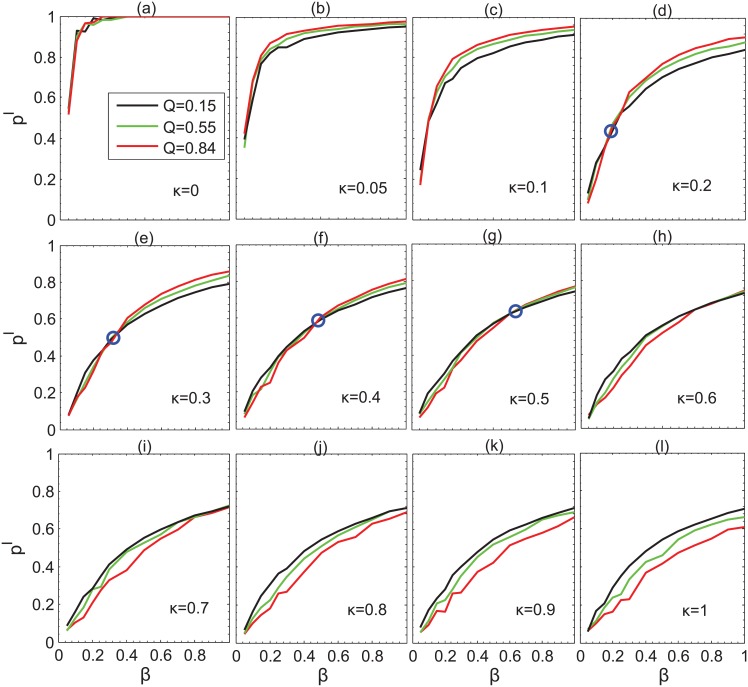
Fraction of final incidence of disease as a function of the infectivity parameter *β*, for different values of parameter *κ*. Other parameters set as: *γ* = 0.2, the community size of epidemic spreading layer ranges from 10 to 96.

By varying the parameter *π*, we implement the epidemic spreading processes on *LFR* networks and the information propagation process on *LFR*−*V* networks with the same network size (*N* = 1000 in our experiments). Note that the communities in the two layers of *LFR*−*V* networks overlap to some extent, as aforementioned. The final incidence of the whole network for different overlap degrees is shown in Fig 10. We can see that for small information diffusion rate, high overlap degree implies high incidence (see the first row of Fig 10). However, the picture is reversed for large diffusion rate, as shown in the bottom row of Fig 10. Interestingly, there is a notable transition from the complete positive correlation between overlap degree and infection incidence to the complete negative correlation in the parameter space of *β*. The blue circle indicates the transition point. It is clear that the transition point emerges at low infectivity rate (corresponding to small value of *β*) and moves to high infectivity rate (corresponding to large value of *β*) as the diffusion process speeds up. This phenomenon roots in the competition between epidemic spreading process and information diffusion. When information diffuses very slowly, clearly high overlap leads to rapid infection. With the increase of diffusion rate, information diffuses faster than epidemic spreading process and therefore the effect of community size appears, which suppresses the propagation of the infection inside the communities. In contrast, low overlap makes the infected nodes have more chance to connect external nodes. Then epidemic spreading process will be less limited by information diffusion, resulting in higher incidence rate.

## Conclusion

Since the advent of network science, the focus of epidemic contagion study has shifted from understanding the emergence and importance of global dynamical properties to the interplay between dynamical processes and network structures. In exploring the topological impacts, communities, at the mesoscopic level of networks, are ubiquitous in many real-world systems and typically play an important role in the dynamic behaviors of a complex system. Our aim in this paper has been to uncover some aspects of the role of communities in epidemic spreading processes.

We modeled the coupled spreading processes by using multiplex network model. We studied the role of community structure in epidemic spreading from several aspects, such as community size, the degree of communities overlap between different layers of a multiplex network. Our results indicate that the diseases awareness obtained through the disease information propagation have effects on epidemic spreading. Particularly, our experiments demonstrate the impact of community feature on epidemic spreading by comparing the dynamics in multiplex networks with community structure and without communities. Furthermore, both the number of communities and the overlap of the communities belonging to different layers have significant influence on disease spreading. These results can be explained by the presence of community size effect on individuals’ response to epidemic spreading and the competition between epidemic spreading process and information diffusion process.
